# Wheat rusts never sleep but neither do sequencers: will pathogenomics transform the way plant diseases are managed?

**DOI:** 10.1186/s13059-015-0615-3

**Published:** 2015-03-02

**Authors:** Lida Derevnina, Richard W Michelmore

**Affiliations:** The Genome Center, University of California, Davis, CA 95616 USA

## Abstract

Field pathogenomics adds highly informative data to surveillance surveys by enabling rapid evaluation of pathogen variability, population structure and host genotype.

## Research highlight

Yellow rust, caused by *Puccinia striiformis* f. sp. *tritici* (PST), is a major disease of wheat and, together with stem rust (*Puccinia graminis*) and leaf rust (*Puccinia triticina*), causes some of the most devastating epidemics on wheat worldwide [1]. Control of these rust pathogens relies predominantly on breeding and deployment of resistant varieties of wheat. To date, nearly 200 wheat-rust-resistance genes have been catalogued [2]; however, resistance has often proved to be ephemeral owing to changes in the pathogen population. In order to increase the durability of resistance, gene-deployment strategies need to consider extant and potential pathogen variability. Although these concepts are not new [3], their implementation was difficult until the advent of high-throughput sequencing (HTS) and genotyping technologies.

Next-generation sequencing technologies provide new opportunities to study pathogens and the hosts they infect. The increasing availability of crop and pathogen genomes [4] is providing new insights into pathogen biology, population structure and pathogenesis. This provides new opportunities for disease management. An important input into resistance breeding programs should be surveillance of the pathogen population. High-throughput pathogenomics offers the possibility for analyzing a large number of pathogen isolates and host varieties rapidly and at low cost.

In an article published in *Genome Biology*, Hubbard and colleagues [5] implemented a robust and rapid method to screen field isolates of PST and their host cultivars. In this particular version of pathogenomics, a selected set of 39 samples of infected wheat and triticale leaf tissue were collected directly from the field in 2013 and analyzed using RNAseq. In addition, the genomes of 21 archived PST isolates from the UK and France were also sequenced. Transcriptome analysis restricted the amount of sequence necessary to obtain diagnostic information for both host and pathogen; this not only accelerated genetic analysis of PST populations *in situ* but also allowed simultaneous assessment of the host genotype in the same sequencing runs. Another advantage of transcriptome analysis is that it detects genes being expressed and therefore the determinants of the interaction; thus, non-expressed genes present in the genome do not obscure genotype-phenotype correlations.

From their analysis of over two million single-nucleotide polymorphisms (SNPs), Hubbard and colleagues discovered that the shift in the 2013 PST population in the UK was due to multiple exotic incursions, which resulted in the dominance of a diverse set of new lineages [5]. The approach was dependent on the availability of draft reference genome assemblies, which enabled the rapid assessment and characterization of variability in the pathogen and host at high resolution [5]. However, a potential limitation of the method is that it only samples at one time-point and does not reveal the genetic potential of non-expressed pathogen genes. Additionally, the authors were able to correlate phenotypic and genotypic data; furthermore, the sequencing data provided greater resolution than phenotyping on resistant cultivars and revealed that some isolates with similar virulence phenotypes were genetically distinct.

## Tracking pathogen variability and movement on a global scale

Pathogenic variation is the underlying cause of the ephemeral efficacy of host resistance genes, with epidemics occurring when new, previously undetected, rust pathotypes emerge possessing new virulence phenotypes [6]. Variation in rust populations has been monitored effectively for many years by using differential sets of host genotypes that express known resistance genes [7]. These surveys have delivered insights into the dynamics of rust populations, including their evolutionary and migratory routes, and have been used extensively to inform breeding programs. Detailed regional, national and international surveys of variability have indicated that, in the absence of sexual recombination, genetic diversity of the rust pathogen population is generated primarily by periodic introduction of exotic isolates, mutation and somatic hybridization [1,8]. However, differential sets have limited genetic resolution, and different sets are used in different regions, making global tracking difficult.

**Figure 1:**
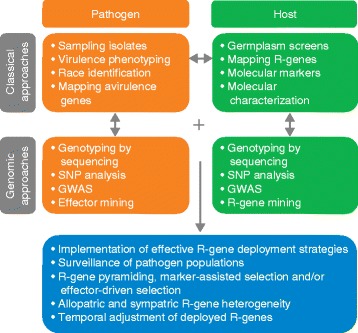
**Summary of the implementation of ‘The Influenza Paradigm’.** In this paradigm [10], deployment of resistance genes is driven by knowledge of pathogen population genomics. Abbreviations: GWAS, genome-wide association studies; R-gene, resistance gene; SNP, single-nucleotide polymorphism.

Field pathogenomics, whether using RNAseq (as described in the paper by Hubbard and colleagues [5]) or genomic DNA-based approaches, will supplement phenotyping (Figure 1). Non-viable samples can be transported to, and sequenced by, regional technology centers, providing standardized information that allows comparative global analysis and monitoring. This is the first step to global coordination of cereal rust surveillance. It is under development for stem rust and is planned for yellow and leaf rusts (http://rusttracker.cimmyt.org). Dense SNP panels will allow tracking of pathogen dispersal on a global scale without the need to move live pathogen samples. Once genotypic variants have been identified, phenotypic characterization can focus on representative samples, optimizing the use of national resources. Unprecedented amounts of information on the genetic structure of rust populations will provide detailed insights into the selective forces driving evolution of new pathotypes. This will allow prediction of which pathotypes are a threat to particular genotypes within specific geographic regions and provide an early-warning system for crop vulnerabilities.

## The contribution of pathogenomics to durable resistance

A major goal in plant breeding is to generate cultivars with durable disease resistance, which remains effective over long periods of time in environments favorable for disease [3]. Several strategies can be employed to enhance the likelihood of durability, one of which is to deploy major genes for resistance in ways that maximize the evolutionary hurdle required of the pathogen to become virulent [3]. A key component for this is an understanding of the determinants of virulence. Rust fungi produce a large arsenal of effector proteins during infection and colonization of the host plant. The re-sequencing of multiple pathogen isolates provides insights into genome organization, plasticity and the presence of pathogenesis-related genes.

Hubbard and colleagues identified 42 genes encoding candidate effectors that shared conserved mutations or expression profiles correlated with virulence profiles and set the stage for downstream functional studies [5]. Sequencing of isolates from multiple geographical areas will reveal the prevalence and distribution of known virulence genes as well as uncover new virulence genes. The identification of genes encoding effector proteins with avirulence activity can discriminate distinct sources of resistance so as to avoid introgression of resistance genes with redundant specificities. However, it should be noted that knowledge of pathogen variation should be coupled with complementary knowledge of variation in the host; this will permit a supply of new effective resistance genes.

Pathogenomics provides the data on host and pathogen needed for deployment of effective resistance genes (Figure 1). This is analogous to the annual adjustment of the viral strains targeted and the distribution of influenza vaccine. High-throughput genotyping that assesses allelic variation in pathogenicity and virulence genes will be important for implementing strategies for the deployment of resistance genes. Continual global monitoring of the pathogen will enable anticipatory strategies that take into consideration the frequency and relative fitness costs of the targeted virulence effectors. Effective combinations of resistance genes can be deployed as pyramids of individual specificities tailored to each region. Continual surveillance of pathogen populations will allow the impact of different resistance genes and strategies to be monitored. This will facilitate rapid responses when resistance breaks down and inform temporal and spatial adjustments in resistance-gene deployment.

## Applicability to other pathosystems

Stem rust, like yellow rust, results in high RNA levels in infected leaves; therefore, the RNAseq approach used by Hubbard and colleagues should be applicable to this and other rust diseases. However, in other pathosystems, the amounts of RNA in infected leaves are much less (for example, 5 to 10% in *Phytophthora infestans* [9]). In these cases, a DNA-based pathogenomics approach might be more appropriate. The ever-increasing efficiency and decreasing costs of sequencing will make pathogenomics at the DNA and RNA levels ever more applicable. Significant increases in sequencing efficiency have already occurred since their study. Automated library construction will allow also an order-of-magnitude more isolates to be sequenced in a timely manner.

## Concluding remarks

‘Rust never sleeps’ was the rallying cry of Nobel Laureate Norman Borlaug, exhorting wheat researchers and breeders to be vigilant against the emergence of new races of rust. Pathogenomics data both document the restlessness of rust pathogens and provide the foundation for putting them to sleep. This, however, requires the use of pathogenomics data in decision-trees informing the rational deployment of resistance genes to achieve durable resistance.

## Abbreviations

HTS: High-throughput sequencing
PST: *Puccinia striiformis* f. sp. *tritici*
SNP: Single-nucleotide polymorphism
